# Live, Attenuated Venezuelan Equine Encephalitis Virus Vaccine (TC83) Causes Persistent Brain Infection in Mice with Non-functional αβ T-Cells

**DOI:** 10.3389/fmicb.2017.00081

**Published:** 2017-01-26

**Authors:** Katherine Taylor, Olga Kolokoltsova, Shannon E. Ronca, Mark Estes, Slobodan Paessler

**Affiliations:** Department of Pathology, University of Texas Medical Branch, GalvestonTX, USA

**Keywords:** VEEV, alphavirus, TC83, immunology, persistent infection

## Abstract

Intranasal infection with vaccine strain of Venezuelan equine encephalitis virus (TC83) caused persistent viral infection in the brains of mice without functional αβ T-cells (αβ-TCR -/-). Remarkably, viral kinetics, host response gene transcripts and symptomatic disease are similar between αβ-TCR -/- and wild-type C57BL/6 (WT) mice during acute phase of infection [0–13 days post-infection (dpi)]. While WT mice clear infectious virus in the brain by 13 dpi, αβ-TCR -/- maintain infectious virus in the brain to 92 dpi. Persistent brain infection in αβ-TCR -/- correlated with inflammatory infiltrates and elevated cytokine protein levels in the brain at later time points. Persistent brain infection of αβ-TCR -/- mice provides a novel model to study prolonged alphaviral infection as well as the effects and biomarkers of long-term viral inflammation in the brain.

## Introduction

Venezuelan equine encephalitis virus (VEEV) is a mosquito-borne arbovirus of the family *Togaviridae*. Evidence for re-emergence of VEEV infections have been identified, although large outbreaks have not been recently observed (Weaver et al., 1996; Rivas et al., 1997). VEEV has a large region where the mosquito vector can be found, setting it as an important emerging infectious disease (Taylor and Paessler, 2013). This is supported by the fact that VEEV is more commonly found in Central and South America than in the United States (Morrison et al., 2008). VEEV’s presence in low-income regions provides the opportunity to develop strategies and improved quality of life to individuals in low-income countries suffering from severe health disparities, as the hours lost to work in these countries severely affect the health and well-being of the entire family structure (Kankeu et al., 2013).

Humans infected with VEEV develop a febrile illness averaging 1–5 days after exposure that can be followed by encephalitis, but asymptomatic infections do occur. The average case fatality rate of VEEV is 1%, but 15% of survivors can be left with severe neurological complications. Currently, there are no licensed vaccines in the United States for use in humans. However, live-attenuated vaccines have been used in the US military and laboratory workers and formalin-inactivated vaccines are available for use in horses.

One such live-attenuated vaccine is TC-83, originally developed by the US Army for vaccine use (Pittman et al., 1996). TC-83 was created by serially passaging the Trinidad Donkey VEEV strain in guinea pig heart cells (Alevizatos et al., 1967). Point mutations in E2 and the 5′ untranslated region are responsible for the attenuated phenotype of TC-83 (Kinney et al., 1993). TC-83 has been noted to be effective in preventing disease in humans, but 15–37.5% of vaccine recipients develop febrile symptoms (Berge et al., 1961; McKinney et al., 1963; Alevizatos et al., 1967; Pittman et al., 1996) and only 82% of vaccinees seroconvert upon vaccination. The probability of plaque reduction neutralization titer remaining ≥1:20 over a period of 5–8 years was 60%. Since TC-83 is only available for use as an investigational vaccine and the population to which it is available is limited, additional studies to evaluate the immunogenicity of the vaccine in humans over time are not available. Of interest to this study is that following intranasal infection with the vaccine strain of VEEV-TC-83, C57BL/6 (WT) mice develop disseminated infection of the brain with high infectious titers without mortality (Hart et al., 1997; Steele et al., 1998; Julander et al., 2008; Taylor et al., 2012).

T cells are an important aspect to the adaptive immune response and control of viral infections. Previously, CD4+ T-cells were demonstrated as crucial in resolution of VEEV-viral brain infection in murine models (Paessler et al., 2007; Yun et al., 2009). Using this knowledge, we tested the hypothesis that T-cells contributed to survival of WT mice following high-level viral replication (TC83 strain) in the brain by evaluating the persistence of virus and the associated cytokine expression between WT and T cell receptor knockout mice.

## Materials and Methods

### Mice and Virus Challenge

Four to 8-week-old female mice with ablated CD4+ and CD8+ T-cell responses due to disruption in T-cell receptor β chain (αβ-TCR -/-) were purchased from Jackson Laboratories (CN# 002118). Four to 8-week-old female wild-type (WT) C57BL6/J mice were purchased from Jackson Laboratories (CN# 000664). All studies were carried out in the ABSL-2 or ABSL-3 as required and were approved by the Institutional Animal Care and Use Committee at the University of Texas Medical Branch. Animals were intranasally infected with 10^7-8^ plaque forming units of TC-83 in 40 μL of saline or saline alone.

### Viral Replication

Organs were homogenized and viral load in the organs was determined by plaque assay on Vero cell monolayers as previously described (Taylor et al., 2012). Statistical analysis and comparison was performed using a two-tailed unpaired student’s *t*-test with 95% confidence interval in GraphPad^®^ Prism (San Diego, CA, USA). In the event the *F* test to compare variance was significantly different, student’s *t*-test was used with Welch’s correction.

Neutralizing antibody was measured by plaque reduction neutralization test as previously described, and geometric mean titers are reported as the reciprocal of the serum dilution corresponding to an endpoint of 50% plaque reduction. For PRNT values below the limit of detection (<20), an arbitrary value of 1 was used for calculation (Taylor et al., 2012).

### Histopathology

Histology of the brain was performed by a blinded neuropathologist as previously described (Taylor et al., 2012). In brief, sections were scored semi-quantitatively using a 0–3 scale. On this scale, 0 represents no inflammation or necrosis, 1 represent mild-minimal inflammation, 2 represents moderate inflammation, and 3 represents moderate/severe inflammation (Taylor et al., 2012).

### Cytokine Analyses

Brain homogenates were utilized to determine cytokine levels. IFN- β levels were determined by ELISA, as per manufacturer’s instructions. Brain homogenate levels of 23 cytokines and chemokines were determined using Bio-Plex Pro Mouse Cytokine 23-plex Assay (Bio-Rad #M60-009RDPD) as per manufacturer’s instructions. Replicates of three mice per group per time point were used. Statistical analysis was performed as described for viral load.

### Microarray Analysis

Total brain RNA preparation, GeneChip processing and microarray analysis were performed as previously described (Taylor et al., 2012). The microarray data from this study can be accessed at http://www.ncbi.nlm.nih.gov/geo/, accession number GSE91074. Transcripts with low probability values adjusted for multiple testing (*p* < 0.0001) and log2 (fold change) > 1 were qualified as differentially expressed.

## Results

### Both Strains of Mice Develop Acute Symptomatic Disease Following TC-83 Intranasal Infection

**FIGURE 1:**
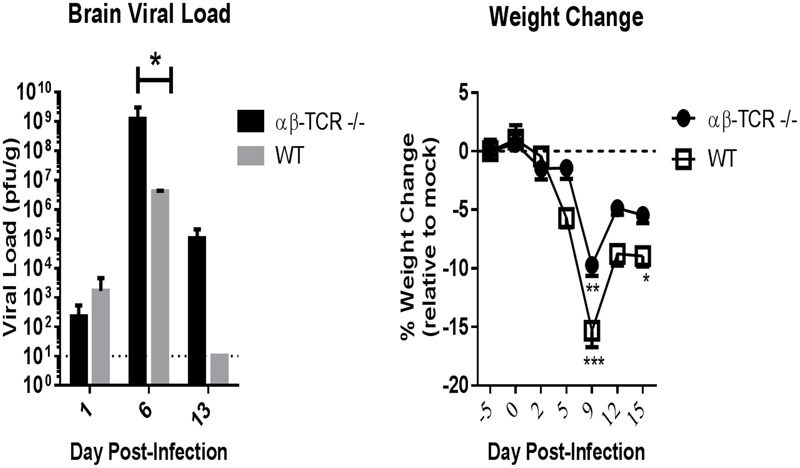
**Mice without functional CD4 and CD8 T-cells (αβ-TCR -/-) and immunocompetent wild-type mice (WT) develop a similar acute phase (D0–D13) viral kinetic and disease response to TC83 infection.** Viral load in the brain as determined by plaque assay at 1, 5, and 13 dpi. αβ-TCR -/- and WT mice display similar viral kinetics over time with viral load peaking at 6 dpi. Combined results of two independent experiments at 6 dpi. *n* = 3 at 1 dpi, *n* = 10 at 6 dpi *n* = 6 at 13 dpi for αβ-TCR -/-; *n* = 3 at 1, 6, and 13 dpi for WT. Mock treated controls (*n* = 3/time point) displayed no virus at any time point. Significance determined by student’s *t*-test *p* < 0.01. Symptomatic disease as measured by weight loss relative to mock controls. αβ-TCR -/- and WT mice display similar weight loss patterns early in disease. *n* = 15 at -5, 0 dpi; *n* = 12 at 2, 5 dpi; *n* = 9 at 9, 12, and 15 dpi. Significance determined by 2-way ANOVA with Sidak’s multiple.

In WT and αβ-TCR -/- mice, infectious virus was initially detected in the brain at 1 dpi (*n* = 3/group), and peak viral loads in the brain occurred at 6 dpi with 100% of tested samples positive. At 6 dpi, the viral burden in the brain of WT mice (4.2 × 10^6^ ± 1.7 × 10^6^ pfu/g) was lower than αβ-TCR-/- mice (1.2 × 10^9^ ± 1.9 × 10^9^ pfu/g) (**Figure 1**). No infectious virus was found in the brains of WT mice past 8 dpi (**Figure 1**). Reduction in brain viral load was observed preposition? αβ-TCR -/- mice between 6 and 13 dpi dropping from a peak of 1.2 × 10^9^ ± 1.9 × 10^9^ pfu/g to 1 × 10^5^ ± 1.1 × 10^5^ pfu/g.

In the peripheral organs, low level of infectious virus (<10^3^ pfu/g) presented in the lung and spleen at 6 dpi only. Infectious virus was detected in the lung in 33% of WT and αβ-TCR -/- mice, and in the spleen in 66% of WT mice only, and no virus was found in the liver in either strain (*n* = 3/group).

Acute symptomatic disease developed following TC-83 infection in both strains of mice. Symptomatic clinical disease was characterized by weight loss. Other clinical signs were rarely observed and limited to piloerection and hunching. Peak weight loss correlated with peak disease in both WT and αβ-TCR -/- mice (**Figure 1**). However, infected WT maintained significantly greater weight loss than mock controls at 15 dpi (**Figure 1**).

### The Acute Host Response in the Brain Is Similar between αβ-TCR -/- and WT Mice at Peak of Infection and Disease

Both αβ-TCR -/- and WT mice developed neutralizing antibody with all serum samples testing positive at 12 dpi (*n* = 8 αβ-TCR -/-, *n* = 9 WT). However, titers were lower in αβ-TCR -/- mice with a range from 40 to 160 with a geometric mean titer of 93 compared to WT with a range from 640 to 2560 with geometric mean titer of 1351 in WT. Interestingly, in αβ-TCR -/- mice reduction of viral load at 13 dpi occurred in the absence of both functional αβ T-cells and significant levels of neutralizing antibody. This indicates that control of infection and disease may be partially attributed to other immune effectors.

In addition to host antibody responses, we contrasted the transcriptional responses of TC83-infected relative mock-infected αβ-TCR -/- and WT mice at the peak of acute disease associated with uniform high viral load in the brain and clinical symptoms, 6 days post-infection. No transcripts simultaneously possess low probability values and high fold change, so none were qualified as differentially expressed. Transcriptome analysis early in infection (at 6 dpi) identified undistinguishable response to intranasal TC-83 inoculation in brains of either WT or αβ TCR -/- mice.

To further clarify the host responses in the brain between αβ-TCR -/- and WT mice, we analyzed 23 cytokine levels in brain homogenates at 1 and 6 dpi.

### Virus Persisted in the Brains of αβ-TCR -/- to 92 dpi

**FIGURE 2:**
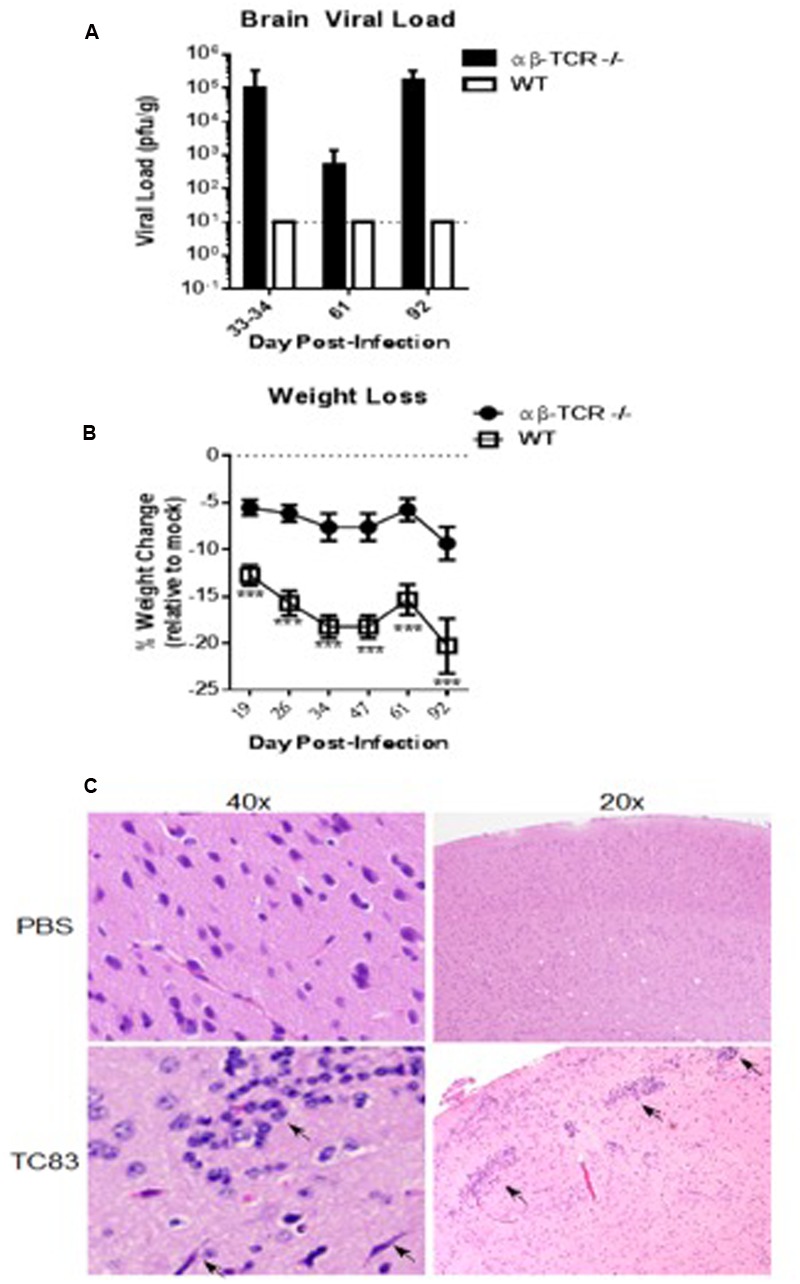
**αβ-TCR -/- mice develop persistent (D13+) viral infection of the brain associated with continued inflammation. (A)** Viral load in the brain as determined by plaque assay. αβ-TCR -/- mice maintain infectious virus in the brain to 92 dpi. Combined results of two independent experiments at 33–34 dpi. *n* = 9 at 33–34 dpi; *n* = 3 at 61, 92 dpi **(B)** Symptomatic disease as measured by weight loss relative to mock controls. By 19 dpi, αβ-TCR -/- display similar weight to mock mice; in contrast, WT mice fail to return to mock treated weight levels at later time points. *n* = 9 at 19, 25 dpi; *n* = 6 at 34, 47, and 61 dpi; *n* = 3 at 92 dpi. **(C)** Characteristic signs of inflammation in persistently infected αβ-TCR -/- mice at 30 dpi including activated microglia, perivascular cuffing, and increased cellular infiltrates. Significance determined by 2-way ANOVA with Sidak’s multiple comparison.

Viral load in the brains of αβ-TCR -/- mice was maintained over time following initial reduction after 6 dpi (**Figure 1**). On 33–34 dpi 60% of tested brain homogenates were positive for infectious virus with brain viral load ranging from undetectable to greater than 10^5^ pfu/g (**Figure 2A**). Only one sample was positive at 61 dpi, but, at 92 dpi, all tested brain samples demonstrated the presence of infectious virus in the brain.

αβ-TCR -/- infected mice returned to a weight similar to mock controls after 19 dpi (**Figure 2B**). Interestingly, WT mice failed to regain lost weight continued to lose weight to 47 and maintained a weight significantly lower than mock controls to end of study (**Figure 2B**).

### A Chronic Pro-inflammatory Cytokine Response in the Brain Characterizes Persistently Infected αβ-TCR -/-

**FIGURE 3:**
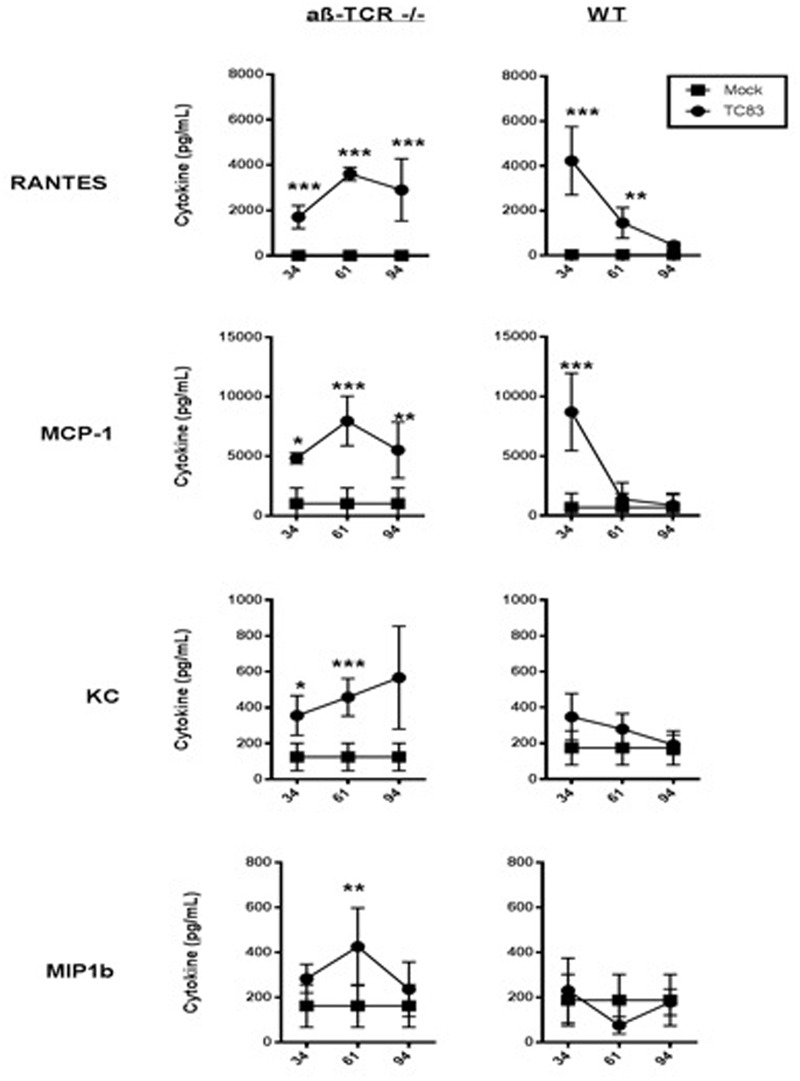
**αβ-TCR -/- mice develop a unique pro-inflammatory cytokine signature that characterizes ongoing inflammation.** Cytokine levels in brain homogenate as determined by multiplex cytokine array at 34, 61, and 92 dpi in αβ-TCR -/- and WT mice. αβ-TCR -/- display increased cytokine levels to 92 dpi. Significance determined by 2-way ANOVA with Sidak’s multiple comparisons. *n* = 3 per group.

A chronic pro-inflammatory cytokine response was observed in αβ-TCR -/- mice. At 34 dpi signs, mononuclear cell infiltrates and meningitis are still apparent αβ-TCR -/- mice (**Figure 2C**). Additionally, neutrophilic infiltrates associated with neuronal necrosis are apparent in the brain tissue at 34 dpi. RANTES and MCP1 are significantly higher in the brains of TC83 infected mice compared to mock αβ-TCR -/- mice at 34, 61, and 92 dpi (**Figure 3**). IFN-β levels also were elevated in persistently infected αβ-TCR -/- mice compared to controls at 6, 13, and 34 dpi. The continued elevation of RANTES and MCP-1 between 34 and 92 dpi in αβ-TCR -/- mice indicates the ongoing infectious processes in the brain, and these cytokines are consistent with the production of antiviral type I interferons.

In contrast to αβ-TCR -/- mice, the cytokine levels in WT mice steadily drop from 34 to 61 dpi returning to baseline by 92 dpi (**Figure 3**).

## Discussion

The novel finding of viral persistence in the brains of αβ-TCR -/- mice combined with indicators of chronic inflammation and/or potential virus persistence below the limit of detection in WT mice requires further study. T-cells appear to be crucial to complete viral clearance and disease resolution in TC83 infection. In absence of functional T-cells and poor neutralizing antibody response, TC83 infection produces higher virus titers in the brain than in brains of wild-type mice. However, higher, chronic brain viral load does not translate to lethal disease indicating that other immune factors apart from antibody or T-cells are responsible for resistance to viral neurological diseases.

The ongoing weight loss in WT mice possibly indicates ongoing pathogenesis with a viral burden below the limit of detection or illness associated with regenerative process in the brain. Although virus was not detectable by plaque assay, this weight loss coincided with ongoing expression of pro inflammatory cytokines in WT mice. The slow decline in cytokine levels could be explained by either regenerative process in the brain after TC83-encephalitis, or continuous presence of virus below the limit of detection.

Chronic infection following alphaviral encephalitis has been reported in the literature in different models (Steele et al., 1998; Hart et al., 2000; Paessler et al., 2007; Brooke et al., 2010). In combination with our model of chronic infection in αβ-TCR -/- mice, data indicates that T-cells are crucial to complete viral clearance and resolution of infection. However, other immune factors may be key in preventing mortality and in allowing the long term asymptomatic, persistence of virus in the brain. We believe that this novel model will be very useful in examining both the process of chronic viral brain infection and the host response to persistent brain infections. It is also noteworthy that the human vaccine TC83 can persist in the brain of αβ-TCR -/- mice without detectable clinical disease, which raises safety concerns regarding the use of the vaccine in human populations and warrant further investigations.

## Author Contributions

All authors listed, have made substantial, direct and intellectual contribution to the work, and approved it for publication.

## Conflict of Interest Statement

The authors declare that the research was conducted in the absence of any commercial or financial relationships that could be construed as a potential conflict of interest.
